# Association between dietary carbohydrate intake and multiple sclerosis risk: a large-scale cohort study

**DOI:** 10.3389/fnut.2025.1654538

**Published:** 2025-10-06

**Authors:** Qi Yuan, Manli Wang, Shuhui Chen, Hao Lin, Yudan Yang, Shuyue Zhao, Baojie Hua, Jing Guo, Xiaohui Sun, Ding Ye, Jiayu Li, Yingying Mao

**Affiliations:** Department of Epidemiology, School of Public Health, Zhejiang Chinese Medical University, Hangzhou, China

**Keywords:** multiple sclerosis, carbohydrates, risk factor, cohort study, UK Biobank

## Abstract

**Introduction:**

Multiple sclerosis (MS) is a chronic autoimmune disorder characterized by neuroinflammation and demyelination. Although diet may influence MS risk, evidence regarding carbohydrate intake remains unclear.

**Methods:**

We examined this association in a prospective cohort of 210,483 participants from the UK Biobank. Dietary carbohydrates were assessed using repeated 24-hour recalls. The diagnosis of MS cases was based on hospital inpatient records coded with the International Classification of Diseases, 10th Revision (ICD-10) code G35. The association between carbohydrate intake and MS risk was analyzed using Cox proportional hazards models.

**Results:**

Over a median follow-up of 13.25 years, 495 incident MS cases were identified. A per interquartile range (IQR) increase in intake of total carbohydrates (HR = 1.21, 95% CI: 1.05–1.40), total sugars (HR = 1.23, 95% CI: 1.10–1.38), fiber (HR = 1.20, 95% CI: 1.08–1.33), fructose (HR = 1.25, 95% CI: 1.12–1.39), and glucose (HR = 1.20, 95% CI: 1.08–1.34) was associated with an increased risk of MS (all false discovery rate [FDR]-*P* < 0.05). Restricted cubic spline analyses showed linear dose–response relationships between these five carbohydrate types and MS risk (all *P_nonlinear_* > 0.05). In addition, the associations between these carbohydrates and MS risk exhibited variations across different age and sex subgroups.

**Discussion:**

Our findings indicate that higher carbohydrate intake is associated with an increased risk of MS. Further studies are warranted to elucidate the underlying mechanisms.

## 1 Introduction

Multiple sclerosis (MS) is an autoimmune disease predominantly mediated by T cells and marked by chronic inflammatory demyelination within the central nervous system (1). The pathophysiology of MS involves disruption of the blood–brain barrier, infiltration of immune cells, and production of myelin-specific autoantibodies, which collectively contribute to axonal damage and subsequent neurological dysfunction (2). Between 2013 and 2020, the global burden of MS increased, with the total number of affected individuals rising from 2.3 million to 2.8 million. In 2020, the estimated global prevalence was 36 per 100,000 population (95% confidence interval [CI]: 35.87–35.95) (3). Notably, the incidence of MS exhibits significant geographic disparities, with European countries reporting the highest rates (4). Given the growing disease burden of MS, conducting etiological research and implementing targeted preventive measures are essential.

Although the precise etiology of MS remains unclear, a growing body of evidence highlights the significant involvement of environmental exposures in MS development (5). Among modifiable risk factors, dietary carbohydrate intake has garnered increasing attention due to its potential role in modulating inflammation, a key mechanism in the onset of MS (6, 7). For instance, refined sugar intake is positively correlated with inflammatory biomarkers, specifically hypersensitive C-reactive protein (hs-CRP) and Interleukin-6 (IL-6), both of which are key players in systemic inflammation (8). Conversely, consumption of dietary fiber and whole grains has been linked to reduced inflammation, a benefit partly attributed to the production of short-chain fatty acids (SCFAs) through microbial fermentation in the gut (9, 10). Moreover, dietary carbohydrates may also have immune-modulating activities. For example, excessive fructose intake can exacerbate symptoms through gut microbiota dysbiosis and immune dysregulation in murine models of experimental autoimmune encephalomyelitis (EAE), which serves as an established animal model for MS (11). Similarly, high glucose levels promote the differentiation of T helper 17 (Th17) cells by activating the mTOR signaling pathway, thereby enhancing autoimmune responses in EAE (12). Collectively, these studies indicate that dietary carbohydrates can modulate both inflammatory and immune pathways in MS, with different types of carbohydrates exerting distinct biological effects.

To date, most nutritional epidemiological studies on MS have focused on overall dietary patterns (13–15), diet quality (16, 17), or specific food groups—such as fish (18), ultra-processed foods (19), fruits and vegetables (19), and processed red meat (20). Research on nutrient intake has largely emphasized fatty acids (21, 22) and vitamin D (23, 24). In contrast, limited population-level evidence exists regarding the role of carbohydrate intake in MS development. Notably, a recent prospective study using the UK Biobank (UKB) examined the association between the intake of grains, staples, vegetables, and fruits—major dietary sources of carbohydrates—and the risk of MS, but found no significant relationship (25). Given that epidemiological evidence on carbohydrates and MS remains scarce, we aimed to address this gap by leveraging the UKB dataset to comprehensively evaluate the associations between total carbohydrate intake, nine specific carbohydrate subtypes, and the risk of MS.

## 2 Methods

### 2.1 Study population

The UKB consists of a large, population-based prospective cohort recruited from individuals across the UK aged 40–69 at baseline. More than 500,000 participants provided genetic samples, biological samples, and completed questionnaires covering demographics, lifestyle factors, and health history. The study was approved by the relevant ethics committee, and informed consent was obtained from all participants prior to their inclusion.

The inclusion and exclusion criteria for this study were as follows: Initially, a total of 502,355 participants were considered. First, we excluded 116 participants who had withdrawn from the study, leaving 502,239 individuals. We then included only those who had completed at least one 24-h dietary recall (*n* = 210,882). Subsequently, participants with a baseline diagnosis of MS were excluded (*n* = 399), yielding a final analytical cohort of 210,483 participants (Supplementary Figure S1).

### 2.2 Outcome determination

The main outcome was the occurrence of newly diagnosed MS cases, identified through hospital inpatient records coded with 10th Revision (ICD-10) code G35 (26). A diagnosis of MS was considered valid if it appeared in either the primary or secondary diagnostic position in any hospital admission record. The date of onset was defined as the earliest recorded diagnosis of MS. The follow-up duration was calculated from the date of the initial assessment until the earliest occurrence of one of the following events: diagnosis of incident MS, death, loss to follow-up, or the end of the study period (31 October 2022).

### 2.3 Exposure definition

Dietary intake data were collected using the Oxford WebQ, a tool specifically developed for large-scale cohort studies. This instrument employs a standardized 24-h dietary recall method to capture detailed consumption information from the previous day, including 206 food items and 32 beverages (27).

Estimates of nutrient intake were derived using the updated version of the Oxford WebQ nutrient calculation system. This method has demonstrated strong validity when benchmarked against calculations based on the UK Food Standards Agency and McCance and Widdowson food composition database. Validation studies indicate a strong agreement between these estimates and dietary intake assessed through interviewer-administered 24-h recalls (energy intake correlation coefficient *r* = 0.96, carbohydrate intake *r* = 0.95) (27, 28).

The UKB collected 24-h dietary recall data over five rounds. The first round was conducted in person at assessment centers between April 2009 and September 2010. Subsequently, eligible participants with valid email addresses were invited to complete four additional online 24-h dietary recalls from February 2011 to June 2012. To enhance the reliability of dietary intake estimates, this study used averaged values from all available dietary assessments (or a single measurement if only one was available) to estimate individual-level carbohydrate consumption (29).

Adjustment for total energy intake was performed using the residual method. Specifically, carbohydrate intake was regressed on total energy intake using linear regression. The residuals from these models were then extracted and combined with the predicted carbohydrate intake at the mean energy intake of the population to derive energy-adjusted carbohydrate variables. These adjusted variables were subsequently used in association analyses with MS. This approach isolates the variation in carbohydrate intake that is independent of total energy intake (30, 31).

### 2.4 Definition and measurement of covariates

Based on existing epidemiological evidence (32–41), the covariates included sociodemographic characteristics, lifestyle factors, and other dietary factors, such as age, sex race, body mass index (BMI), education, Townsend deprivation index (TDI), smoking status, alcohol drinking status, physical activity, energy-adjusted fat, and energy-adjusted protein. Participants with a college, university, or professional degree, as well as those with a vocational qualification, were classified as having a “High” level of education. Those who had completed A-levels, AS-levels, O-levels, GCSEs, CSEs, or equivalent qualifications were categorized as having a “Medium” level of education. All other participants were classified as having a “Low” level of education. Socioeconomic status was quantified using the continuous TDI, derived from UK Office for National Statistics area-level deprivation data linked to the residential postcodes of participants. Regular physical activity was defined as fulfilling at least one of the following criteria: accumulating 150 min or more of moderate-intensity exercise per week; 75 min or more of vigorous-intensity exercise per week; performing moderate-intensity activity on five or more days per week; or engaging in vigorous-intensity activity on at least 1 day per week (42).

### 2.5 Statistical analysis

All statistical analyses were performed using R software (version 4.2.3). Responses marked as “Prefer not to answer” or “Do not know” were treated as missing data. To enhance data completeness and ensure the robustness of the results, multiple imputation was applied to handle missing covariate data (43). The imputation models were selected based on the variable type (continuous, binary, or ordinal), and the resulting imputed datasets were used in subsequent analyses. Cox proportional hazards regression models were employed to evaluate the association between energy-adjusted carbohydrate intake and the risk of MS. To examine potential linear associations, restricted cubic splines were fitted, using the 25th, 50th, and 75th percentiles of carbohydrate intake as knot locations. Additionally, stratified analyses were conducted by sex (men vs. women) and by age group (< 60 years vs. ≥ 60 years).

The robustness of the results was evaluated through a series of sensitivity analyses: (1) exclusion of participants with missing covariate data; (2) removal of individuals diagnosed with MS within the first two years after baseline; (3) omission of participants with implausible energy intake (for men: < 3,347 kJ/day or > 17,573 kJ/day; for women: < 2,092 kJ/day or > 14,644 kJ/day) (44); (4) restriction to participants who completed at least three dietary assessments; (5) additional adjustment for type 2 diabetes, hyperlipidemia, and hypertension (45, 46); (6) further adjustment for energy-adjusted vitamin D intake (47, 48); (7) exclusion of individuals with any of the 16 pre-existing autoimmune diseases (e.g., rheumatoid arthritis, celiac disease) at baseline (49), with the corresponding ICD-10 codes provided in Supplementary Table S1.

## 3 Results

Of the 210,483 individuals free of MS at enrollment, 495 developed the disease over a median follow-up of 13.25 years (interquartile range [IQR]: 12.67–14.05). Among these incident cases, 358 (72.3%) were female. Table 1 presents the baseline demographic characteristics and macronutrient intakes across quartiles (Q1-Q4) of total carbohydrate intake. With increasing carbohydrate consumption, the proportions of male participants and individuals engaging in physical activity showed a gradual upward trend. Intakes of energy, fat, and protein also increased across the quartiles. Age was remarkably consistent across all four groups. The remaining characteristics did not follow a strict monotonic increasing or decreasing trend across quartiles but instead exhibited some variation.

**Table 1:** Baseline characteristics across the lowest and highest quartiles of total carbohydrate intake.

Characteristics	Quartiles of total carbohydrates (g/d)			
	Q1 (≤201.94)	Q2 (>201.94 and ≤ 247.24)	Q3 (>247.24 and ≤ 298.05)	Q4 (>298.05)
*N*	52,621	52,621	52,620	52,621
Age (y), median (IQR)	57.00 (12.00)	57.00 (13.00)	57.00 (13.00)	57.00 (14.00)
Sex, *n* (%)				
Female	35,776 (67.99)	32,343 (61.46)	27,538 (52.33)	20,220 (38.43)
Male	16,845 (32.01)	20,278 (38.54)	25,082 (47.67)	32,401 (61.57)
Race, *n* (%)				
White	49,667 (94.39)	50,569 (96.10)	50,702 (96.35)	49,842 (94.72)
Others	2,762 (5.25)	1866 (3.55)	1732 (3.29)	2,574 (4.89)
Missing	192 (0.36)	186 (0.35)	186 (0.35)	205 (0.39)
TDI, median (IQR)	−2.17 (4.04)	−2.37 (3.84)	−2.40 (3.66)	−2.27 (3.84)
BMI, kg/m^2^, median (IQR)	26.48 (5.84)	26.13 (5.52)	26.09 (5.44)	26.35 (5.49)
Education, *n* (%)				
High	26,251 (49.89)	28,117 (53.44)	28,751 (54.63)	28,379 (53.93)
Medium	20,836 (39.60)	20,043 (38.09)	19,645 (37.33)	19,382 (36.83)
Low	5,172 (9.83)	4,214 (8.01)	4,035 (7.67)	4,618 (8.78)
Missing	362 (0.69)	247 (0.47)	189 (0.36)	242 (0.46)
Alcohol drinking status, *n* (%)				
Current	49,414 (93.91)	49,580 (94.22)	49,435 (93.95)	48,575 (92.31)
Previous	1,491 (2.83)	1,455 (2.77)	1,536 (2.92)	1943 (3.69)
Never	1,656 (3.15)	1,529 (2.91)	1,609 (3.06)	2054 (3.90)
Missing	60 (0.11)	57 (0.11)	40 (0.08)	49 (0.09)
Smoking status, *n* (%)				
Current	5,029 (9.56)	3,806 (7.23)	3,450 (6.56)	4,197 (7.98)
Previous	19,443 (36.95)	18,888 (35.89)	18,307 (34.79)	18,040 (34.28)
Never	27,981 (53.17)	29,794 (56.62)	30,742 (58.42)	30,243 (57.47)
Missing	168 (0.32)	133 (0.25)	121 (0.23)	141 (0.27)
Physical activity, *n* (%)				
No	14,956 (28.42)	14,090 (26.78)	13,051 (24.80)	11,610 (22.06)
Yes	37,009 (70.33)	38,047 (72.30)	39,153 (74.41)	40,505 (76.97)
Missing	656 (1.25)	484 (0.92)	416 (0.79)	506 (0.96)
Energy, kJ/d, median (IQR)	6242.94 (1842.28)	7701.96 (1554.64)	8900.52 (1642.29)	10991.40 (2605.61)
Fat (g/d), median (IQR)	52.45 (26.92)	64.34 (26.39)	73.98 (28.63)	91.58 (38.05)
Protein (g/d), median (IQR)	65.68 (24.96)	74.62 (22.48)	81.49 (23.28)	94.52 (29.86)
Total sugars (g/d), median (IQR)	80.41 (34.10)	108.76 (32.62)	132.07 (37.08)	170.71 (57.7)
Free sugar (g/d), median (IQR)	33.11 (25.89)	48.26 (28.62)	62.20 (33.72)	86.37 (50.04)
Starch (g/d), median (IQR)	83.62 (37.06)	115.66 (32.86)	138.00 (36.85)	174.31 (56.27)
Fiber (g/d), median (IQR)	12.31 (5.78)	16.10 (5.68)	18.57 (6.18)	22.56 (8.45)
Fructose (g/d), median (IQR)	17.93 (12.78)	24.55 (13.90)	29.23 (15.66)	36.45 (21.27)
Glucose (g/d), median (IQR)	16.56 (10.58)	22.82 (11.36)	27.44 (12.89)	34.72 (18.02)
Lactose (g/d), median (IQR)	9.98 (8.07)	12.59 (8.40)	14.27 (8.89)	16.51 (10.50)
Maltose (g/d), median (IQR)	2.83 (2.72)	4.10 (3.25)	5.21 (3.99)	7.42 (6.45)
Sucrose (g/d), median (IQR)	27.22 (16.45)	38.48 (17.99)	48.21 (21.41)	65.88 (33.83)

BMI, body mass index; CI, confidence interval; HR, hazard ratio; IQR, interquartile range; TDI, Townsend deprivation index.

The distribution of carbohydrate and its subtypes in both MS and non-MS populations is presented in Supplementary Table S2. Compared with the non-MS group, individuals with MS had significantly higher energy-adjusted intakes of carbohydrates (median: 260.65 *vs.* 256.01 g/d, *p* = 0.018), total sugars (median: 128.18 *vs.* 122.78 g/d, *p* < 0.001), fiber (median: 17.72 *vs.* 17.39 g/d, *p* = 0.012), fructose (median: 28.48 *vs.* 26.69 g/d, *p* < 0.001), glucose (median: 26.24 *vs.* 25.11 g/d, *p* = 0.004), and sucrose (median: 47.49 *vs.* 45.20 g/d, *p* = 0.001).

Associations of MS risk with each IQR increase in energy-adjusted carbohydrate intake are shown in Table 2. In the age- and sex-adjusted model (Model 1), per IQR increase in intake of energy-adjusted total carbohydrates (HR = 1.15, 95% CI: 1.03–1.28), total sugars (1.19, 1.08–1.31), fiber (1.14, 1.03–1.26), fructose (1.17, 1.06–1.29), glucose (1.15, 1.04–1.26), and sucrose (1.14, 1.04–1.25) were positively associated with MS risk, and all associations remained significant after false discovery rate (FDR) correction (all FDR-*p* < 0.05). After further adjustment for sociodemographic and lifestyle factors (Model 2), we observed that the associations for these carbohydrate types with MS risk were either slightly strengthened or remained similar, and all remained statistically significant (all FDR-*p* < 0.05). In the fully adjusted model, which included energy-adjusted fat and protein intake (Model 3), total carbohydrates (HR = 1.21, 95% CI: 1.05–1.40), total sugars (1.23, 1.10–1.38), fiber (1.20, 1.08–1.33), fructose (1.25, 1.12–1.39), and glucose (1.20, 1.08–1.34) continued to show positive associations with MS risk (all FDR-*p* < 0.05), with no substantial changes in effect sizes compared to Model 2. However, energy-adjusted sucrose was no longer statistically significant after FDR correction (FDR-*p* = 0.052).

**Table 2:** Association between per IQR increase in carbohydrate intake and the risk of multiple sclerosis.

Energy-adjusted carbohydrate intake (per IQR increase)	Model 1			Model 2			Model 3		
	HR (95%CI)	*p*	FDR-*P*	HR (95%CI)	*p*	FDR-*P*	HR (95%CI)	*p*	FDR-*P*
Total carbohydrates	**1.15 (1.03–1.28)**	**0.012**	**0.020**	**1.21 (1.08–1.35)**	**0.001**	**0.001**	**1.21 (1.05–1.40)**	**0.008**	**0.015**
Total sugars	**1.19 (1.08–1.31)**	**<0.001**	**0.004**	**1.24 (1.12–1.37)**	**<0.001**	**<0.001**	**1.23 (1.10–1.38)**	**<0.001**	**0.001**
Free sugar	1.08 (0.98–1.19)	0.134	0.179	1.07 (0.97–1.18)	0.172	0.229	1.02 (0.92–1.14)	0.659	0.718
Starch	0.96 (0.86–1.07)	0.416	0.499	0.97 (0.87–1.08)	0.614	0.687	0.95 (0.85–1.06)	0.321	0.385
Fiber	**1.14 (1.03–1.26)**	**0.011**	**0.020**	**1.19 (1.08–1.32)**	**0.001**	**0.001**	**1.20 (1.08–1.33)**	**0.001**	**0.002**
Fructose	**1.17 (1.06–1.29)**	**0.002**	**0.008**	**1.24 (1.13–1.37)**	**<0.001**	**<0.001**	**1.25 (1.12–1.39)**	**<0.001**	**0.001**
Glucose	**1.15 (1.04–1.26)**	**0.007**	**0.016**	**1.21 (1.09–1.33)**	**<0.001**	**0.001**	**1.20 (1.08–1.34)**	**0.001**	**0.003**
Lactose	0.98 (0.88–1.09)	0.718	0.783	0.98 (0.88–1.10)	0.750	0.750	0.99 (0.89–1.11)	0.894	0.894
Maltose	1.00 (0.93–1.07)	0.964	0.964	0.98 (0.92–1.05)	0.630	0.687	0.96 (0.90–1.03)	0.259	0.345
Sucrose	**1.14 (1.04–1.25)**	**0.005**	**0.016**	**1.14 (1.04–1.25)**	**0.005**	**0.009**	**1.11 (1.01–1.22)**	**0.030**	0.052

Cases/total person-years: 495/2770945. Model 1, adjusted for age and sex. Model 2, model 1 adjustments plus adjusted for race, education, TDI, BMI, smoking status, alcohol drinking status, and physical activity. Model 3, model 2 adjustments plus energy-adjusted fat and energy-adjusted protein. BMI, body mass index; CI, confidence interval; HR, hazard ratio; IQR, interquartile range; TDI, Townsend deprivation index. Bold values indicate statistically significant results (*p* < 0.05 or FDR-*p* < 0.05) and HRs (95% CIs) showing statistical significance.

In addition to analyzing carbohydrate intake as a continuous variable, we examined the associations between the five aforementioned carbohydrate types and MS by categorizing their intake into quartiles (Q1–Q4). As shown in Supplementary Table S3, participants in the third quartile (Q3) of energy-adjusted total carbohydrate intake exhibited a significantly higher MS risk compared to those in the lowest quartile (Q1) (HR = 1.41, 95% CI: 1.08–1.84) in the fully adjusted model. Energy-adjusted total sugars also demonstrated a positive association (Q4 vs. Q1: HR = 1.71, 95% CI: 1.29–2.27). Higher fiber intake corresponded to increased MS risk (Q4 vs. Q1: HR = 1.43, 95% CI: 1.10–1.86), and similar positive associations emerged for fructose (Q4 vs. Q1: HR = 1.62, 95% CI: 1.22–2.15) and glucose (Q4 vs. Q1: HR = 1.40, 95% CI: 1.06–1.86). These quartile-based findings align with the per IQR increase analysis, reinforcing a positive link between carbohydrate intake and MS risk.

We conducted several sensitivity analyses and found that the majority of the results remained stable, with minimal changes in effect sizes and sustained statistical significance. Notably, when the analysis was restricted to participants who completed at least three 24-h dietary assessments, the association between dietary fiber and MS was of borderline statistical significance, while the HR remained largely unchanged (HR = 1.19, 95% CI: 1.00–1.41; *p* = 0.056). For other carbohydrate types (total carbohydrates, total sugars, fructose, and glucose), greater variability in effect estimates was observed in this sensitivity analysis, although statistical significance was largely maintained. This increased variability may be attributed to the substantial reduction in sample size resulting from the inclusion criterion of at least three dietary assessments (Supplementary Table S4).

**Figure 1:**
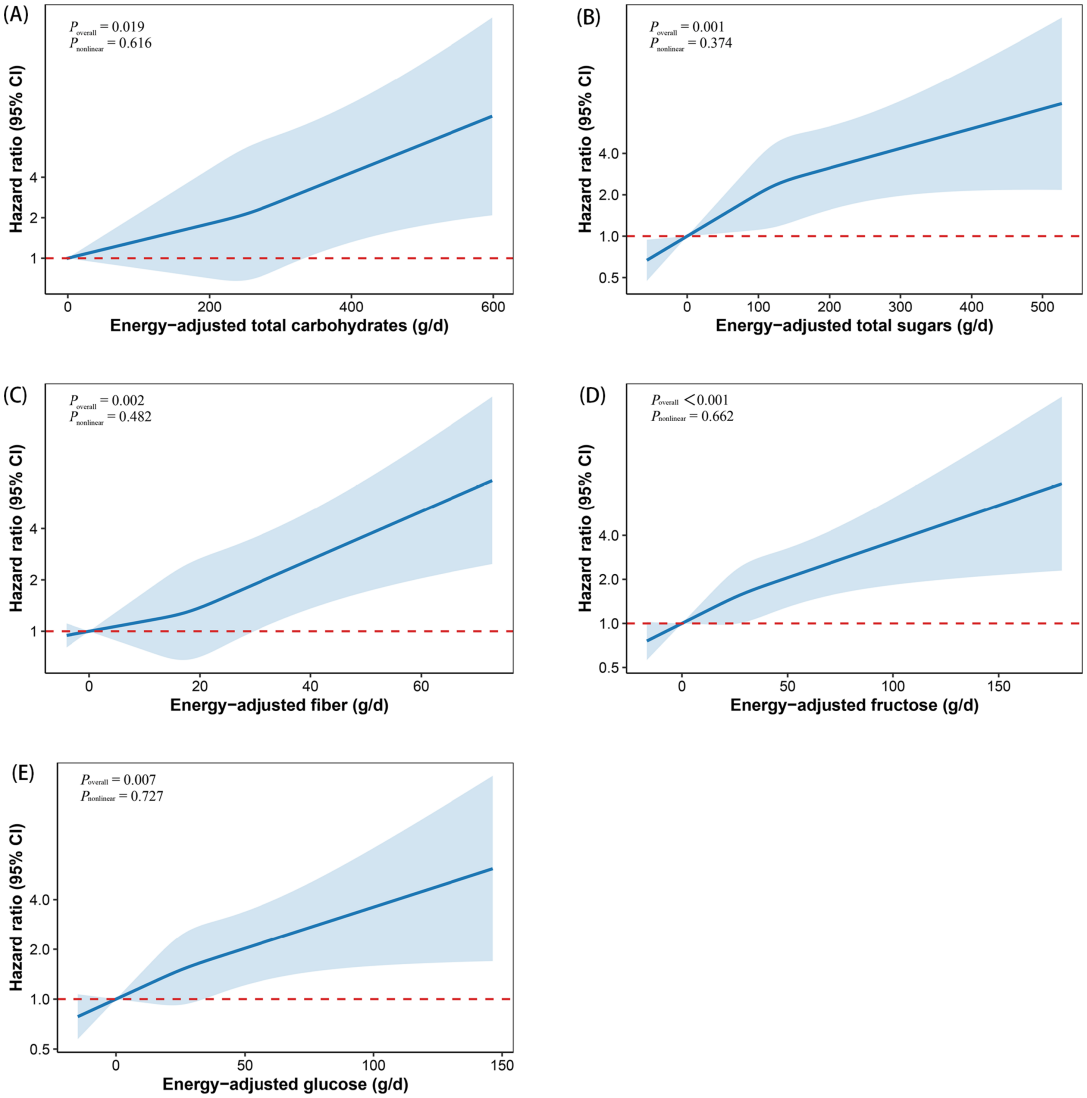
Restricted cubic spline curves depicting the dose–response relationship between total carbohydrates **(A)**, total sugars **(B)**, fiber **(C)**, fructose **(D)**, and glucose **(E)** and the risk of multiple sclerosis. Models were adjusted for age, sex, race, education, BMI, TDI, smoking status, alcohol drinking status, physical activity, energy-adjusted fat, and energy-adjusted protein. The x-axis represents carbohydrate intake, excluding extreme values at the 5th and 95th percentiles. The hazard ratio is shown as a solid line, with the corresponding 95% confidence interval represented by the shaded area between the dashed lines. BMI, body mass index; CI, confidence interval; TDI, Townsend deprivation index.

Based on these findings, we plotted RCS curves to visualize the dose–response relationships between carbohydrate intake and MS risk, as shown in Figure 1. Total carbohydrates, total sugars, fiber, fructose, and glucose intake all showed positive associations with MS risk (all *P _overall_* < 0.05). Furthermore, each relationship was found to be linear (all *P _nonlinear_* > 0.05).

**Figure 2:**
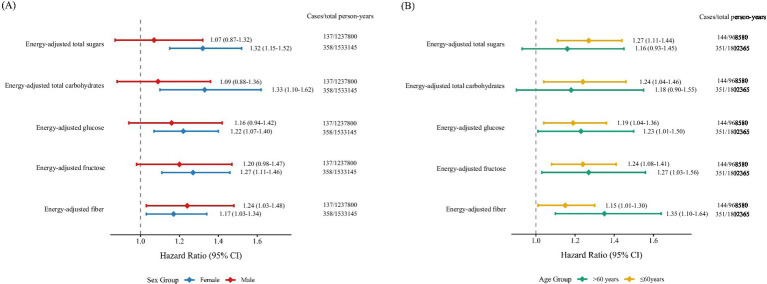
Forest plots of subgroup analyses illustrating the association between carbohydrate intake and multiple sclerosis risk, stratified by **(A)** sex and **(B)** age. Models were adjusted for age, sex, race, education, BMI, TDI, smoking status, alcohol drinking status, physical activity, energy-adjusted fat, and energy-adjusted protein. BMI, body mass index; CI, confidence interval; HR, hazard ratio; TDI, Townsend deprivation index.

The stratified analysis revealed that the associations of total carbohydrates, total sugars, fiber, fructose, and glucose with MS remained statistically significant only in females, but not in males. In age-stratified analyses, significant associations were observed for total carbohydrates and total sugars only among participants younger than 60 years old, whereas no significant associations were found in those aged 60 or older. In contrast, the strength of the associations for fiber, fructose, and glucose with MS appeared to be stronger in the older age group (≥60 years) (Figure 2).

## 4 Discussion

Leveraging data from the UKB, this prospective cohort study provides the comprehensive evidence linking dietary carbohydrate intake and its subclasses with incident MS risk. Higher intakes of total carbohydrates, total sugars, fiber, fructose, and glucose were significantly associated with increased MS risk, with dose–response analyses revealing a linear association between these carbohydrate intake and MS incidence. Furthermore, these associations exhibited variations by sex and age.

Our findings indicate that a higher intake of carbohydrates is associated with an increased risk of MS, consistent with earlier reports. For example, the participants with MS were found to consume more carbohydrates than healthy individuals in a previous case–control study (50). Similarly, following a Mediterranean diet—which is naturally low in refined carbohydrates—has been linked to a lower risk of MS (51). Intervention studies further support the importance of carbohydrate quality: substituting refined grains with whole grains such as rye and whole-wheat pasta was shown to reduce levels of IL-6, an inflammatory molecule implicated in MS (52). Collectively, these findings suggest that both the quantity and quality of carbohydrates may influence MS risk.

In addition to total carbohydrate intake, we found that high sugar intake was significantly associated with increased MS risk. This aligns with mechanistic insights from a animal study, which indicated that excessive sugar may disrupt gut bacteria, particularly by depleting butyrate-producing bacteria that are crucial for maintaining both intestinal and blood–brain barrier integrity (53). Such dysbiosis could contribute to neuroinflammation and immune dysregulation, potentially explaining our observations. In particular, fructose and glucose—simple sugars abundant in sweetened foods and beverages—were strongly associated with MS risk. Experimental studies have shown that chronic high-fructose intake can induce hippocampal neuroinflammation, gliosis, and neuronal loss in mice (54), while glucose has been shown to promote Th17 cell differentiation, a process known to exacerbate neuroinflammatory responses (12). Nevertheless, further human studies are needed to clarify the specific roles of different sugars in MS pathogenesis.

From a public health perspective, our study highlights the potential of dietary modification as a population-wide strategy for reducing the risk of MS. The observed association between the intake of carbohydrates and sugars—particularly fructose and glucose—and MS incidence suggests that nutritional interventions could contribute meaningfully to the primary prevention of MS. These findings are consistent with new WHO guidelines strongly recommending that adults and children limit their daily intake of free sugars (defined as monosaccharides and disaccharides added to foods and beverages) to less than 10% of total energy intake to lower the risk of overweight, obesity, and dental caries (55). By extending the relevance of sugar reduction to neurological health, our results support the integration of dietary recommendations into public health policies aimed at preventing chronic immune-mediated diseases.

Interestingly, although fiber is generally considered beneficial due to its anti-inflammatory and gut-health promoting properties, our results showed a positive association with MS risk. This unexpected finding may be due to the different types of fiber. Dietary fibers are commonly classified into soluble and insoluble types based on their water solubility. Soluble fibers—such as inulin and guar gum—can be fermented by colonic microbiota, yielding metabolites including short-chain fatty acids like butyrate (56). In contrast, insoluble fibers, such as cellulose and hemicellulose, primarily enhance intestinal motility and are less readily utilized by gut microbes (57). Notably, emerging evidence suggests that different types of fiber may have divergent effects on health. For instance, previous study has reported that high doses of soluble fibers may promote colorectal carcinogenesis in mice, while insoluble fibers did not show similar effects (58). More specifically, high intake of soluble fiber may induce gut microbiota dysbiosis—enriching potential pathogens and reducing beneficial commensals—thereby facilitating colorectal tumorigenesis. Additionally, soluble fiber can disrupt intestinal metabolism, leading to elevated fecal butyrate and serum bile acids, along with reduced fecal inosine. Although direct evidence linking soluble fiber to MS is limited, we hypothesized that the positive association observed in our study may reflect high intake of soluble fiber and subsequent microbial metabolite activity, which could influence MS pathogenesis through the gut–brain axis. This hypothesis warrants further experimental validation.

Stratified analysis by sex revealed that the significant association between carbohydrate intake and MS was observed only in females. From a statistical perspective, this may be attributed to the larger sample size in this subgroup compared to males, where the limited sample size might have resulted in insufficient statistical power to detect a significant HR. Furthermore, the observed differences in associations may also reflect underlying sex-specific physiological mechanisms. MS incidence is higher in women, particularly during reproductive age (20–49 years), with a female-to-male ratio reaching up to 3:1 (59, 60). Hormonal influences play a key role in immune modulation. Estrogen, exhibits a biphasic immunomodulatory effect: it stimulates immune responses at low concentrations but acts as a strong immunosuppressant during pregnancy (33). Moreover, women generally demonstrate stronger antibody and T cell-mediated immune responses, which further contribute to this sexual dimorphism (61). The inherently higher susceptibility of women to MS may explain why the observed positive association between carbohydrate intake and MS was more pronounced in females.

Our study has some limitations should be noted. First, the UKB cohort primarily comprises middle-aged and older White participants, which limits generalizability to early-onset MS and other populations. Second, residual confounding cannot be completely excluded, particularly with respect to factors such as gut microbiome composition, which warrants prioritization in future research. Third, despite the prospective design, lag-period analysis, and extensive covariate adjustment, reverse causality remains plausible. Individuals with early MS symptoms or a high-risk profile may have altered their dietary habits prior to enrollment. However, the robustness of our findings after excluding early cases and adjusting for baseline chronic conditions mitigates this concern. Fourth, dietary data were self-reported, introducing potential measurement error. Fifth, although the overall cohort was large, the number of incident MS cases was modest, limiting statistical power and highlighting the need for replication in larger cohorts. Sixth, the UKB does not distinguish fiber subtypes (e.g., soluble vs. insoluble), preventing clarification of whether the observed positive association with MS risk is driven primarily by soluble fiber. Finally, while this study identifies correlative associations, it does not provide experimental validation of the mechanisms by which dietary carbohydrates influence MS pathology, which is a crucial next step that we plan to undertake in subsequent research.

## 5 Conclusion

In conclusion, we found significant associations between higher intakes of total carbohydrates, total sugars, fiber, fructose, and glucose and an increased MS risk. These findings offer valuable insights for developing dietary interventions focused on carbohydrate regulation to mitigate MS risk, which may help reduce its public health burden.

## Data Availability

Publicly available datasets were analyzed in this study. This data can be found at: UK Biobank (https://www.ukbiobank.ac.uk/), Application Number: 113769.

## Supplementary material

SUPPLEMENTARY Table S2 *p* values < 0.05 are shown in bold.

SUPPLEMENTARY Table S3 HRs (95% CIs) that are statistically significant are shown in bold.

SUPPLEMENTARY Table S4 HRs (95% CIs) showing statistical significance and *p* < 0.05 are also shown in bold.

## References

[1] Oh J Vidal-Jordana A Montalban X. Multiple sclerosis: clinical aspects. Curr Opin Neurol. (2018) 31:752–9. doi: 10.1097/wco.0000000000000622, PMID: 30300239

[2] Dendrou CA Fugger L Friese MA. Immunopathology of multiple sclerosis. Nat Rev Immunol. (2015) 15:545–58. doi: 10.1038/nri3871 26250739

[3] Federation MSI. Atlas of Ms 3rd edition (2020). Available online at: https://atlasofms.org/map/global/epidemiology/number-of-people-with-ms. (Accessed June 10, 2025).

[4] Walton C King R Rechtman L Kaye W Leray E Marrie RA. Rising prevalence of multiple sclerosis worldwide: insights from the atlas of Ms, third edition. Mult Scler. (2020) 26:1816–21. doi: 10.1177/1352458520970841, PMID: 33174475 PMC7720355

[5] Filippi M Bar-Or A Piehl F Preziosa P Solari A Vukusic S. Multiple Sclerosis. Nat Rev Dis Primers. (2018) 4:43. doi: 10.1038/s41572-018-0041-4 30410033

[6] Basu A Devaraj S Jialal I. Dietary factors that promote or retard inflammation. Arterioscler Thromb Vasc Biol. (2006) 26:995–1001. doi: 10.1161/01.ATV.0000214295.86079.d1, PMID: 16484595

[7] Muth AK Park SQ. The impact of dietary macronutrient intake on cognitive function and the brain. Clin Nutr. (2021) 40:3999–4010. doi: 10.1016/j.clnu.2021.04.043, PMID: 34139473

[8] Esposito K Marfella R Ciotola M Di Palo C Giugliano F Giugliano G. Effect of a Mediterranean-style diet on endothelial dysfunction and markers of vascular inflammation in the metabolic syndrome: a randomized trial. JAMA. (2004) 292:1440–6. doi: 10.1001/jama.292.12.1440 15383514

[9] Cronin P Joyce SA O’Toole PW O’Connor EM. Dietary fibre modulates the gut microbiota. Nutrients. (2021) 13:655. doi: 10.3390/nu13051655, PMID: 34068353 PMC8153313

[10] Reynolds A Mann J Cummings J Winter N Mete E Te Morenga L. Carbohydrate quality and human health: a series of systematic reviews and Meta-analyses. Lancet. (2019) 393:434–45. doi: 10.1016/s0140-6736(18)31809-9, PMID: 30638909

[11] Peterson SR Ali S Shrode RL Mangalam AK. Effect of a fructose-rich diet on gut microbiota and immunomodulation: potential factors for multiple sclerosis. Immunohorizons. (2023) 7:213–27. doi: 10.4049/immunohorizons.2300008, PMID: 36939622 PMC10240966

[12] Zhang D Jin W Wu R Li J Park SA Tu E. High glucose intake exacerbates autoimmunity through reactive-oxygen-species-mediated Tgf-Β cytokine activation. Immunity. (2019) 51:671–81.e5. doi: 10.1016/j.immuni.2019.08.001, PMID: 31451397 PMC9811990

[13] Keykhaei F Norouzy S Froughipour M Nematy M Saeidi M Jarahi L. Adherence to healthy dietary pattern is associated with lower risk of multiple sclerosis. J Cent Nerv Syst Dis. (2022) 14:11795735221092516. doi: 10.1177/11795735221092516, PMID: 35558004 PMC9087291

[14] Noormohammadi M Ghorbani Z Naser Moghadasi A Saeedirad Z Shahemi S Ghanaatgar M. Mind diet adherence might be associated with a reduced odds of multiple sclerosis: results from a case-control study. Neurol Ther. (2022) 11:397–412. doi: 10.1007/s40120-022-00325-z, PMID: 35094301 PMC8857348

[15] Sedaghat F Jessri M Behrooz M Mirghotbi M Rashidkhani B. Mediterranean diet adherence and risk of multiple sclerosis: a case-control study. Asia Pac J Clin Nutr. (2016) 25:377–84. doi: 10.6133/apjcn.2016.25.2.12, PMID: 27222422

[16] Mannino A Lithander FE Dunlop E Hoare S Shivappa N Daly A. A Proinflammatory diet is associated with an increased likelihood of first clinical diagnosis of central nervous system demyelination in women. Mult Scler Relat Disord. (2022) 57:103428. doi: 10.1016/j.msard.2021.103428, PMID: 34856497

[17] Pommerich UM Nielsen R Overvad K Dahm CC Tjønneland A Olsen A. Diet quality is not associated with late-onset multiple sclerosis risk—a Danish cohort study. Mult Scler Relat Disord. (2020) 40:101968. doi: 10.1016/j.msard.2020.101968, PMID: 32035368

[18] Rezaeizadeh H Mohammadpour Z Bitarafan S Harirchian MH Ghadimi M Homayon IA. Dietary fish intake and the risk of multiple sclerosis: a systematic review and Meta-analysis of observational studies. Nutr Neurosci. (2022) 25:681–9. doi: 10.1080/1028415x.2020.1804096, PMID: 32787642

[19] Mannino A Daly A Dunlop E Probst Y Ponsonby AL van der Mei IAF. Higher consumption of ultra-processed foods and increased likelihood of central nervous system demyelination in a case-control study of Australian adults. Eur J Clin Nutr. (2023) 77:611–4. doi: 10.1038/s41430-023-01271-1, PMID: 36754977 PMC10169648

[20] Black LJ Bowe GS Pereira G Lucas RM Dear K van der Mei I. Higher non-processed red meat consumption is associated with a reduced risk of central nervous system demyelination. Front Neurol. (2019) 10:125. doi: 10.3389/fneur.2019.00125, PMID: 30837942 PMC6389668

[21] Gu J Bao Y Li Y Hua L Deng X Zhang Y. Dietary N-6 polyunsaturated fatty acid intake and brain health in middle-aged and elderly adults. Nutrients. (2024) 16:272. doi: 10.3390/nu16244272, PMID: 39770894 PMC11680004

[22] Bjørnevik K Chitnis T Ascherio A Munger KL. Polyunsaturated fatty acids and the risk of multiple sclerosis. Mult Scler. (2017) 23:1830–8. doi: 10.1177/1352458517691150, PMID: 28156186 PMC5494026

[23] Munger KL Zhang SM O’Reilly E Hernán MA Olek MJ Willett WC. Vitamin D intake and incidence of multiple sclerosis. Neurology. (2004) 62:60–5. doi: 10.1212/01.wnl.0000101723.79681.38 14718698

[24] Munger KL Chitnis T Frazier AL Giovannucci E Spiegelman D Ascherio A. Dietary intake of vitamin D during adolescence and risk of multiple sclerosis. J Neurol. (2011) 258:479–85. doi: 10.1007/s00415-010-5783-1, PMID: 20945071 PMC3077931

[25] Barbero Mazzucca C Scotti L Comi C Vecchio D Chiocchetti A Cappellano G. The role of diet in multiple sclerosis onset: a prospective study using Uk biobank. Nutrients. (2024) 16:746. doi: 10.3390/nu16111746, PMID: 38892680 PMC11174354

[26] World Health Organization. International statistical classification of diseases and related health problems. 10th revision, Fifth edition, 2016 ed. Geneva: World Health Organization (2015).

[27] Liu B Young H Crowe FL Benson VS Spencer EA Key TJ. Development and evaluation of the Oxford Webq, a low-cost, web-based method for assessment of previous 24 H dietary intakes in large-scale prospective studies. Public Health Nutr. (2011) 14:1998–2005. doi: 10.1017/s1368980011000942, PMID: 21729481

[28] Perez-Cornago A Pollard Z Young H van Uden M Andrews C Piernas C. Description of the updated nutrition calculation of the Oxford Webq questionnaire and comparison with the previous version among 207,144 participants in Uk biobank. Eur J Nutr. (2021) 60:4019–30. doi: 10.1007/s00394-021-02558-4, PMID: 33956230 PMC8437868

[29] Greenwood DC Hardie LJ Frost GS Alwan NA Bradbury KE Carter M. Validation of the Oxford Webq online 24-hour dietary questionnaire using biomarkers. Am J Epidemiol. (2019) 188:1858–67. doi: 10.1093/aje/kwz165, PMID: 31318012 PMC7254925

[30] Willett W Stampfer MJ. Total energy intake: implications for epidemiologic analyses. Am J Epidemiol. (1986) 124:17–27. doi: 10.1093/oxfordjournals.aje.a114366, PMID: 3521261

[31] McCullough LE Byrd DA. Total energy intake: implications for epidemiologic analyses. Am J Epidemiol. (2023) 192:1801–5. doi: 10.1093/aje/kwac071, PMID: 35419586

[32] Goyne CE Fair AE Sumowski PE Graves JS. The impact of aging on multiple sclerosis. Curr Neurol Neurosci Rep. (2024) 24:83–93. doi: 10.1007/s11910-024-01333-2, PMID: 38416310

[33] Krysko KM Graves JS Dobson R Altintas A Amato MP Bernard J. Sex effects across the lifespan in women with multiple sclerosis. Ther Adv Neurol Disord. (2020) 13:1756286420936166. doi: 10.1177/1756286420936166, PMID: 32655689 PMC7331774

[34] Milo R Kahana E. Multiple sclerosis: Geoepidemiology, genetics and the environment. Autoimmun Rev. (2010) 9:A387–94. doi: 10.1016/j.autrev.2009.11.010, PMID: 19932200

[35] Liu Z Zhang TT Yu J Liu YL Qi SF Zhao JJ. Excess body weight during childhood and adolescence is associated with the risk of multiple sclerosis: a Meta-analysis. Neuroepidemiology. (2016) 47:103–8. doi: 10.1159/000450854, PMID: 27723651

[36] Zeng R Jiang R Huang W Wang J Zhang L Ma Y. Dissecting shared genetic architecture between obesity and multiple sclerosis. EBioMedicine. (2023) 93:104647. doi: 10.1016/j.ebiom.2023.104647, PMID: 37300932 PMC10363440

[37] Wu J Olsson T Hillert J Alfredsson L Hedström AK. Influence of Oral tobacco versus smoking on multiple sclerosis disease activity and progression. J Neurol Neurosurg Psychiatry. (2023) 94:589–96. doi: 10.1136/jnnp-2022-330848, PMID: 37001984 PMC10359558

[38] Kleerekooper I Chua S Foster PJ Trip SA Plant GT Petzold A. Associations of alcohol consumption and smoking with disease risk and neurodegeneration in individuals with multiple sclerosis in the United Kingdom. JAMA Netw Open. (2022) 5:e220902. doi: 10.1001/jamanetworkopen.2022.0902, PMID: 35238934 PMC8895260

[39] Hedström AK Olsson T Alfredsson L. Smoking is a major preventable risk factor for multiple sclerosis. Mult Scler. (2016) 22:1021–6. doi: 10.1177/1352458515609794, PMID: 26459151

[40] Bjørnevik K Riise T Cortese M Holmøy T Kampman MT Magalhaes S. Level of education and multiple sclerosis risk after adjustment for known risk factors: the Envims study. Mult Scler. (2016) 22:104–11. doi: 10.1177/1352458515579444, PMID: 26014605 PMC4702243

[41] Olsson T Barcellos LF Alfredsson L. Interactions between genetic, lifestyle and environmental risk factors for multiple sclerosis. Nat Rev Neurol. (2017) 13:25–36. doi: 10.1038/nrneurol.2016.187 27934854

[42] World Health Organization. Who guidelines on physical activity and sedentary behaviour Geneva: World Health Organization (2020) Available online at: https://iris.who.int/handle/10665/336656 (Accessed 20 May 2025).

[43] Azur MJ Stuart EA Frangakis C Leaf PJ. Multiple imputation by chained equations: what is it and how does it work? Int J Methods Psychiatr Res. (2011) 20:40–9. doi: 10.1002/mpr.329, PMID: 21499542 PMC3074241

[44] Willett W. Nutritional epidemiology. New York: Oxford University Press (2012).

[45] Greenwood DC Threapleton DE Evans CE Cleghorn CL Nykjaer C Woodhead C. Glycemic index, glycemic load, carbohydrates, and type 2 diabetes: systematic review and dose-response Meta-analysis of prospective studies. Diabetes Care. (2013) 36:4166–71. doi: 10.2337/dc13-0325, PMID: 24265366 PMC3836142

[46] Shah M Adams-Huet B Bantle JP Henry RR Griver KA Raatz SK. Effect of a high-carbohydrate versus a high--*cis*-monounsaturated fat diet on blood pressure in patients with type 2 diabetes. Diabetes Care. (2005) 28:2607–12. doi: 10.2337/diacare.28.11.2607, PMID: 16249527

[47] Bagur MJ Murcia MA Jiménez-Monreal AM Tur JA Bibiloni MM Alonso GL. Influence of diet in multiple sclerosis: a systematic review. Adv Nutr. (2017) 8:463–72. doi: 10.3945/an.116.014191, PMID: 28507011 PMC5421121

[48] Jin D Wu S Zhang YG Lu R Xia Y Dong H. Lack of vitamin D receptor causes Dysbiosis and changes the functions of the murine intestinal microbiome. Clin Ther. (2015) 37:996–1009.e7. doi: 10.1016/j.clinthera.2015.04.004, PMID: 26046242

[49] He MM Lo CH Wang K Polychronidis G Wang L Zhong R. Immune-mediated diseases associated with Cancer risks. JAMA Oncol. (2022) 8:209–19. doi: 10.1001/jamaoncol.2021.5680, PMID: 34854871 PMC8640951

[50] Machado SB Cabral R Murade N Ares NC Scorcine C Fragoso YD. Dietary habits in a Group of Patients with multiple sclerosis are similar to those of healthy control subjects. Arq Neuropsiquiatr. (2020) 78:638–41. doi: 10.1590/0004-282x20200065, PMID: 33146289

[51] Alfredsson L Olsson T Hedström AK. Inverse association between Mediterranean diet and risk of multiple sclerosis. Mult Scler. (2023) 29:1118–25. doi: 10.1177/13524585231181841, PMID: 37366345

[52] Stampanoni Bassi M Iezzi E Drulovic J Pekmezovic T Gilio L Furlan R. Il-6 in the cerebrospinal fluid signals disease activity in multiple sclerosis. Front Cell Neurosci. (2020) 14:120. doi: 10.3389/fncel.2020.00120, PMID: 32655367 PMC7324533

[53] Kawano Y Edwards M Huang Y Bilate AM Araujo LP Tanoue T. Microbiota imbalance induced by dietary sugar disrupts immune-mediated protection from metabolic syndrome. Cell. (2022) 185:3501–19.e20. doi: 10.1016/j.cell.2022.08.005, PMID: 36041436 PMC9556172

[54] Li JM Yu R Zhang LP Wen SY Wang SJ Zhang XY. Dietary fructose-induced gut Dysbiosis promotes mouse hippocampal Neuroinflammation: a benefit of short-chain fatty acids. Microbiome. (2019) 7:98. doi: 10.1186/s40168-019-0713-7, PMID: 31255176 PMC6599330

[55] World Health Organization. Who calls on countries to reduce sugars intake among adults and children (2015) Available online at: https://www.who.int/news/item/04-03-2015-who-calls-on-countries-to-reduce-sugars-intake-among-adults-and-children (Accessed 29 August 2025).

[56] Guan ZW Yu EZ Feng Q. Soluble dietary Fiber, one of the Most important nutrients for the gut microbiota. Molecules. (2021) 26:802. doi: 10.3390/molecules26226802, PMID: 34833893 PMC8624670

[57] Npv P Joye IJ. Dietary fibre from whole grains and their benefits on metabolic health. Nutrients. (2020) 12:3045. doi: 10.3390/nu12103045 33027944 PMC7599874

[58] Yang J Wei H Lin Y Chu ESH Zhou Y Gou H. High soluble Fiber promotes colorectal tumorigenesis through modulating gut microbiota and metabolites in mice. Gastroenterology. (2024) 166:323–37.e7. doi: 10.1053/j.gastro.2023.10.012, PMID: 37858797

[59] Gold SM Willing A Leypoldt F Paul F Friese MA. Sex differences in autoimmune disorders of the central nervous system. Semin Immunopathol. (2019) 41:177–88. doi: 10.1007/s00281-018-0723-8 30361800

[60] Orton SM Herrera BM Yee IM Valdar W Ramagopalan SV Sadovnick AD. Sex ratio of multiple sclerosis in Canada: a longitudinal study. Lancet Neurol. (2006) 5:932–6. doi: 10.1016/s1474-4422(06)70581-6, PMID: 17052660

[61] Whitacre CC. Sex differences in autoimmune disease. Nat Immunol. (2001) 2:777–80. doi: 10.1038/ni0901-777, PMID: 11526384

